# Autologous Biologic Treatment with Fat, Bone Marrow Aspirate and Platelet Rich Plasma Is an Effective Alternative to Total Knee Arthroplasty for Patients with Moderate Knee Arthrosis

**DOI:** 10.3390/medicines7060037

**Published:** 2020-06-25

**Authors:** Chadwick Prodromos, Susan Finkle

**Affiliations:** Illinois Sportsmedicine and Orthopaedic Centers, Glenview, IL 60025, USA; suef@ismoc.net

**Keywords:** bone marrow aspirate, adipose tissue, PRP, osteoarthritis, stem cell, autologous

## Abstract

**Background:** Osteoarthrosis (OA) of the knee afflicts millions worldwide. Total Knee Arthroplasty (TKA) is common, but associated with substantial cost and morbidity. Prior studies of intra-articular injection of fat, bone marrow aspirate (BMA), and platelet rich plasma (PRP) have shown clinical benefit. We hypothesized that injection of autologous adipose tissue, BMA, and PRP would provide significant benefit for patients with moderate knee OA resulting in avoidance of total knee arthroplasty (TKA) in most, with discontinuance of NSAIDs and other drugs. **Methods:** 42 TKA candidate patients (47 knees) with moderate (Kellgren-Lawrence 2 and 3) knee OA who had failed conservative treatment had autologous adipose tissue, BMA, and PRP injection as an alternative to TKA in office using only local anesthetic. Patients had discontinuance of all nonsteroidal anti-inflammatory medicines (NSAIDs) and other analgesics, except acetaminophen, prior to treatment. Patients were evaluated with Knee injury and Osteoarthritis Outcome Score Physical Shortform (KOOS-PS), Western Ontario and McMaster Universities Osteoarthritis Index (WOMAC), and Single Assessment Numeric Evaluation (SANE) prior to treatment, and at 6 months, 1, and 2 years after treatment. **Results:** Follow up exceeded 80% at all time points. There were no significant adverse events. TKA was avoided in 97% at one and 86% at two years after treatment. Mean SANE, KOOS-PS, and WOMAC scores significantly improved at 6 months, 1, and 2 years post-treatment. WOMAC and SANE scores were higher at two versus one year post-treatment. **Conclusions:** Combined fat, BMA, and PRP injection is a safe and effective treatment for moderate knee OA, with reliable avoidance of TKA and possible continued improvement at two year follow-up.

## 1. Introduction

Osteoarthrosis of the knee affects hundreds of millions of people worldwide, and results in tremendous pain, suffering, and economic loss [1]. Until now, there has been no effective treatment for knee arthrosis. Corticosteroid injections are widely used but damage cartilage [2,3], predispose to often devastating infection after TKA [4,5], are often associated with severe complications, such as stress fracture and avascular necrosis [6,7,8,9], and have been shown to accelerate arthrosis and increase the incidence of TKA [2,9]. Nonsteroidal anti-inflammatory medicines (NSAID) drugs are also widely used, but have high toxicity rates [10]. Aweid [11] reported that chronic NSAID use for treatment of hip and knee osteoarthritis may have a higher mortality rate than joint replacement surgery. In our experience, they also tend to accelerate the path to TKA by masking pain, which allows patients to damage their joints more rapidly than they otherwise would. Hyaluronic acid helps some patients, but studies have shown its efficacy to be marginal. Multiple injections are often necessary at each treatment cycle, and it must generally be repeated every six months [12]. There has been no effective non-surgical treatment, so total knee arthroplasty (TKA) is now performed extensively for OA, but is associated with substantial cost and morbidity [12,13,14,15,16]. The incidence of TKA has increased dramatically in recent years, and is now commonly performed for even mild and moderate arthrosis [14,17,18]. 

There is thus a great need for a safe, effective, non-surgical treatment for knee arthrosis. Multiple studies have demonstrated that treatment with adipose tissue and its derivatives results in improvement in osteoarthritis symptoms in both the knee [19,20,21,22] and in the hip [23]. Other studies have shown similar results with bone marrow aspirate [24,25,26,27,28,29], and with a combination of bone marrow aspirate and adipose tissue [30,31]. Platelet rich plasma (PRP) has been shown to also improve arthritis symptoms [32,33]. All of these treatments have been shown to be very safe and effective for the treatment of osteoarthritis. We therefore hypothesized that injection of a combination of fat, bone marrow aspirate and PRP would produce significant clinical benefit for knee OA, would be very safe, and would produce greater duration of benefit than has been shown with other injection treatments. We also hypothesized that it would allow avoidance of TKA in TKA-candidate patients.

## 2. Materials and Methods 

We prospectively evaluated all patients with moderate osteoarthritis who received combined autologous adipose tissue, bone marrow, and PRP injections into one or both of their knees from September 2015 to March 2019. All patients had Kellgren Lawrence scores of 2 or 3, had failed conservative treatment for osteoarthritis, and were candidates for TKA. Patients were excluded from treatment if they had an active infection, active malignancy, or inflammatory arthritis. If patients were using anticoagulants, they were asked to continue their use before, during, and after treatment. All subjects gave their informed consent for inclusion before they participated in the study. The study was conducted in accordance with the Declaration of Helsinki, and the protocol was approved by the Ethics Committee of the Foundation for Orthopaedics and Regenerative Medicine (#2016-02, 26 March 2020).

### 2.1. Preparation of the Injections

#### 2.1.1. Bone Marrow

Patients were positioned prone on a treatment table. After sterile-preparation and the application of local anesthetic, a Marrow Cellution (Ranfac Corp) bone marrow aspiration needle was used to harvest 10 cc of bone marrow from the posterior ipsilateral iliac crest, following the manufacturer’s instructions. A sampling technique was used so that each milliliter was aspirated from a slightly different location to increase the yield of mesenchymal stem cells [34]. 

#### 2.1.2. Autologous Adipose Tissue

After a bandage was applied to the bone marrow harvest incision, patients were repositioned on their side. After sterile-preparation, tumescence (saline solution containing lidocaine and epinephrine) was injected subcutaneously into the fatty areas of the waist/hip/buttock region, depending on individual fat distribution. Tissue and tumescence were harvested into syringes, washed with 250 cc of saline, gently spun (1300 rpm for 3 min) and sized to 1.2 mm using sizing transfers (Tulip Medical) to produce 9 cc of adipose tissue. 

#### 2.1.3. PRP

A total of 45 cc of blood was drawn and processed through a double spin technique to create 4 mL of PRP. According the PAW classification system, the PRP preparation was P3-Aα [35].

### 2.2. Treatment Regimen 

After sterile preparation, the three preparations were injected sequentially into the knee joint using separate syringes and a single 18 gauge needle held with a clamp. Patients were instructed to restrict activity for the first week to activities of daily living regarding their lower extremities. Ice and acetaminophen were allowed to treat pain, especially during the first 24 to 48 h. After one week, patients were allowed to resume any non-painful activity. One month after initial treatment, most patients received a second PRP injection to boost response to the initial treatment. 

### 2.3. Outcome Data

Patients were asked to discontinue all nonsteroidal anti-inflammatory medicines (NSAIDs) and other analgesics except for acetaminophen before treatment. Prior to initial treatment, each patient completed a Knee injury and Osteoarthritis Outcome Score Physical Shortform (KOOS-PS), a Western Ontario and McMaster Universities Osteoarthritis Index (WOMAC), and Single Assessment Numeric Evaluation (SANE) assessment. 

Clinical outcome and pain post treatment were evaluated using the KOOS-PS, WOMAC, and SANE assessments at 6 months, 1 year, and 2 years after treatment. Patients were also evaluated for global improvement by asking: “What percent better or worse are you now compared to before treatment”. The outcome was considered significantly improved if patients stated they were overall at least 25% improved compared to their status before treatment. This rating differs from the VAS rating in that it was designed to allow the patient to factor in function and motion, as well as pain when judging their post treatment status. 

## 3. Results

Forty-three patients (48 knees) received treatment. One patient fell 5 months after treatment, required knee surgery and was excluded from the study, leaving 42 patients and 47 knees. There were 19 male and 23 female patients ranging in age from 46 to 88 years (mean age = 67). Mean BMI was 28.4. Two patients were using daily low dose aspirin, and one was anti-coagulated with apixaban. Thirty seven of the 47 joints (79%) received a second PRP injection 1 month after the initial treatment. Additional PRP injections were performed at subsequent time periods based on patient outcomes and desires. Seven joints received one additional PRP injection beyond the 1 month period (four did not receive the 1 month injection), ranging in time from 3 months to 37 months after the initial treatment (median time 6 months). Five joints received two additional PRP injections beyond the 1 month period (two of these did not get a PRP injection at 1 month) with a median time to the first injection post treatment of 4 months. Three joints received four additional injections with a median time of 12 months post treatment to the first injection. The remaining 32 patients did not receive additional PRP injections, although three of them received 1 HA injection at 11 months post treatment.

No patients had any adverse event after treatment other than expected self-limited soreness and/or swelling at harvest and injection sites. The use of ice and/or acetaminophen was recommended for the first 2 days if needed. None of the three patients using anti-coagulants had hematoma, excessive bruising, or other problems after the treatment. No abdominal binders or special dressings were used or needed after the lipo-aspiration.

**Figure 1:**
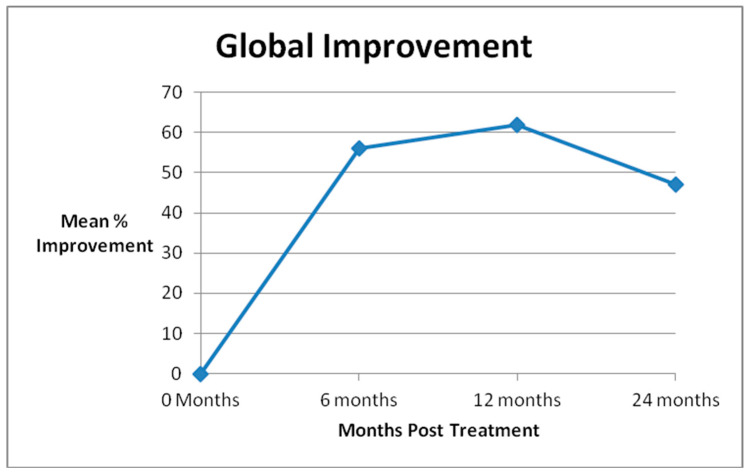
Mean Global Improvement.

Forty-one of 47 knees (87%) had 6 month follow-up, 38 of 47 knees (81%) had 1 year follow-up, and 22 of 26 knees (85%) had 2 year follow-up. Significant improvement, as measured by the global improvement score, was achieved by 80% of patients at 6 months, 76% at 1 year, and 68% at 2 years after treatment. The global improvement score mean magnitude of improvement was 56% at 6 months, 62% at 1 year, and 47% at 2 years after treatment (Figure 1). Outcome scores are summarized in Table 1. 

**Figure 2:**
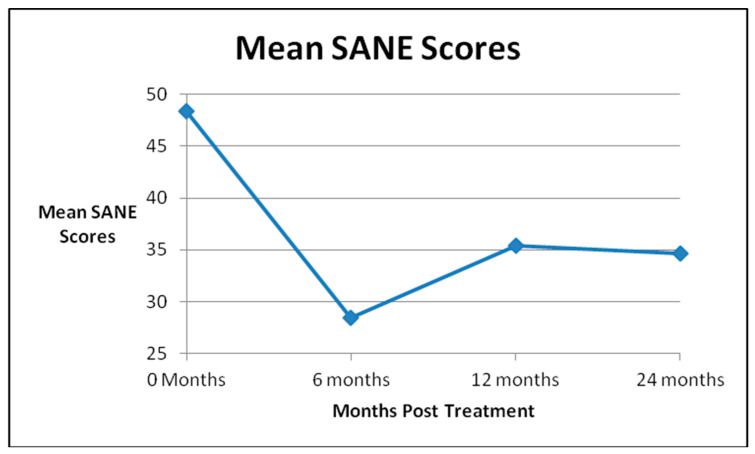
Single Assessment Numeric Evaluation (SANE) Scores.

**Figure 3:**
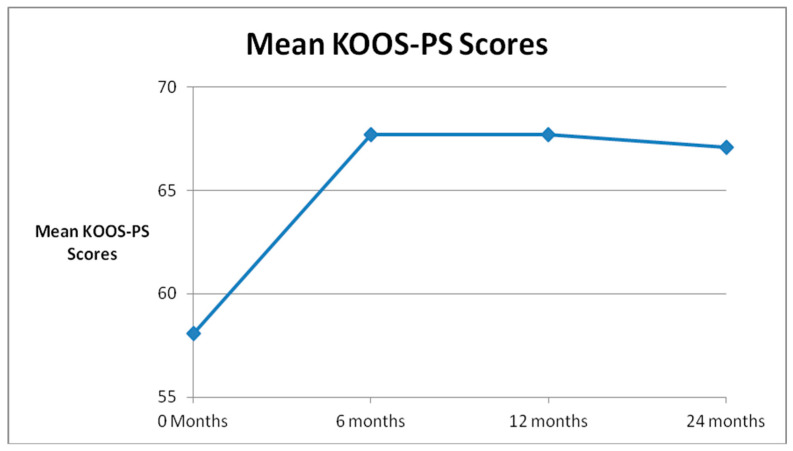
Mean Knee injury and Osteoarthritis Outcome Score Physical Shortform (KOOS-PS) Scores.

**Figure 4:**
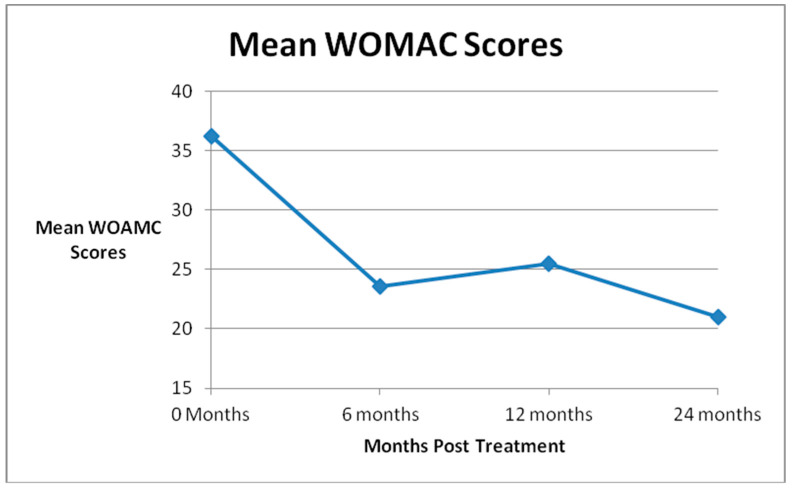
Mean Western Ontario and McMaster Universities Osteoarthritis Index (WOMAC) Scores.

Mean SANE scores (0–100, 100 worst) for all patients demonstrated statistically significant improvements from the pre-injection score of 48.4 to all follow up time points: 29.1 at 6 months, 36.3 at 1 year, and 34.6 at 2 years. (See Figure 2 The mean KOOS-PS scores (0–100, 0 worst) improved significantly from 58.1 for all patients before treatment to 66.8 at 6 months, 66.7 at 1 year, and 67.1 at 2 years. (See Figure 3 The mean WOMAC scores (0–96, 96 worst) declined significantly from 36.2 for all patients before treatment to 24.3 at 6 months, 26.4 at 1 year and 21.0 at 2 years. (Figure 4)The results were statistically significant for all outcome scores at all follow up points. The *p* value can be found in Table 1. 

Three patients had total knee arthroplasty during the follow-up period. One was performed 9 months after treatment, and two were performed between 13 and 24 months. The TKA avoidance rate was 97% at one year post treatment and 86% at two years.

Patients were sorted into age cohorts (age <60, age 60–69, age 70–79, age 80). No differences in outcomes were seen between the four groups. Additionally, patients were sorted into three cohorts based on BMI; “normal weight” with a BMI of less than 25, “overweight” with a BMI of 25 to less than 29.9, and “obese” with a BMI of 30 or more. No differences in outcomes were seen between these three groups. 

## 4. Discussion

This study shows significant and sustained improvement in moderate osteoarthritis symptoms in patients treated with combined autologous adipose tissue, bone marrow, and PRP injections. These results are similar to the results seen in a study by Centeno, which also used a combination of bone marrow, fat, and PRP [30]. However, the follow up in Centeno’s study was an average of 10.7 months. This study extends the results out to two years, and shows continued improvement through this period, including sustained improvement in KOOS-PS scores, and a trend toward increasing improvement in WOMAC scores and SANE scores at two years compared to one year. 

A significant strength of the study is the high rate of follow-up and the statistical significance of the results. Follow up was reported here at two years, however, these patients are still being followed, so that we will be able to provide more data in the future as to how long benefit from a single stem cell injection is likely to last.

One of the most important findings was the high rate of successful avoidance of knee replacement two years post treatment. The primary goals of treatment were to allow patients to live a good lifestyle, discontinue analgesic and anti-inflammatory drugs, and avoid knee replacement surgery. These goals were achieved in the overwhelming majority.

Another important finding of this study is that neither age nor BMI had any significant effect on the outcome. This is the first study to our knowledge to address the effects of age and BMI on the results of stem cell treatment and it is notable that no effect was seen. Both hyaluronic acid injections and TKA have been shown to be less effective in older and heavier patients [36,37,38,39,40]. Dangerous adverse events are more common during and after TKA in older and heavier patients [36,38,39]. Therefore, the autologous biologic treatment provided in this study would appear to offer a safer and more effective alternative for older and heavier patients. The complete absence of significant morbidity makes this particularly appealing for these patients. 

Elderly patients are often told that their stem cells are not suitable for use and are ineffective due to their age. Often this argument is used to convince elderly patients to be treated with allogeneic biologic treatment or joint replacement surgery. The results of this study show that this is not the case. Tissue from older patients performed just as well as tissue from younger patients. The results of treatment for patients in their ninth decade of life were quite good, and no different than for the younger patients in our series. This is important, because autologous stem cell treatment is safer and less expensive than allogeneic cellular treatment and joint replacement surgery. Our study clearly shows that recommending against autologous treatment for these older patients is unwarranted.

It is notable that in this study, even full anti-coagulation, which is sometimes needed in older patients, did not produce adverse events. Patients were specifically directed to not interrupt their anticoagulation regimen to avoid risk. This differs from surgical intervention, where patients must discontinue anti-coagulants for several days, to decrease the risks of a bleeding complication. Similarly, the lack of sedation during this treatment avoided any medication side effects. Only local anesthesia was used.

PRP was used to help augment the effect of the stem cells. It proved a useful “booster”, since none of the study patients needed repeat stem cell injection. It was largely used on an as needed basis, although most patients received one at the one month visit after their stem cell injection. This one month booster PRP was not performed in some patients, due to scheduling issues. The variability of this regimen does represent a weakness of this study for the purposes of data analysis. However, we believe, the overall effect of the stem cell treatment is still quite clear, and we provided PRP where needed, as our first obligation is the treatment of our patients. The larger point is that with occasional PRP as needed, stem cell treatment does not need to be repeated for many years. 

The study combined fat, bone marrow and PRP. Thus, another weakness of the study is that it is impossible to know which was the most important. We have begun a regimen using only fat and PRP, which may help answer this question.

## 5. Conclusions

The combination of fat, bone marrow aspirate and PRP is a safe and effective office procedure for the treatment of moderate knee arthrosis. It provides reliable avoidance of knee replacement surgery at two year follow-up. Improvement may increase from the first to second year after treatment. Neither advancing age nor higher BMI adversely affected outcome. 

## Figures and Tables

**Table 1:** Outcome Scores.

	Global Improvement		SANE Scores			KOOS-PS Scores			WOMAC Scores		
	#	Mean % Improvement	#	Mean Score	*p* Value	#	Mean Score	*p* Value	#	Mean Score	*p* Value
**Pre Treatment**			46	48.4		45	58.1		33	36.2	
**6 Month FU** **(N = 45)**	41	56	41	28.4	*p* = 0.0002	37	67.7	*p* = 0.0004	37	23.6	*p* = 0.002
**1 Year FU** **(N = 45)**	38	62	37	35.4	*p* = 0.02	32	67.7	*p* = 0.004	31	25.5	*p* = 0.02
**2 Years FU** **(N = 31)**	22	47	18	34.6	*p* = 0.05	19	67.1	*p* = 0.02	20	21.0	*p* = 0.002

#—Number of joints.
